# Supplementary figures and images for: A computational model of liver tissue damage and repair
Source: PLoS One. 2020 Dec 21;15(12):e0243451. doi: 10.1371/journal.pone.0243451 (PMC7752149; doi:10.1371/journal.pone.0243451)

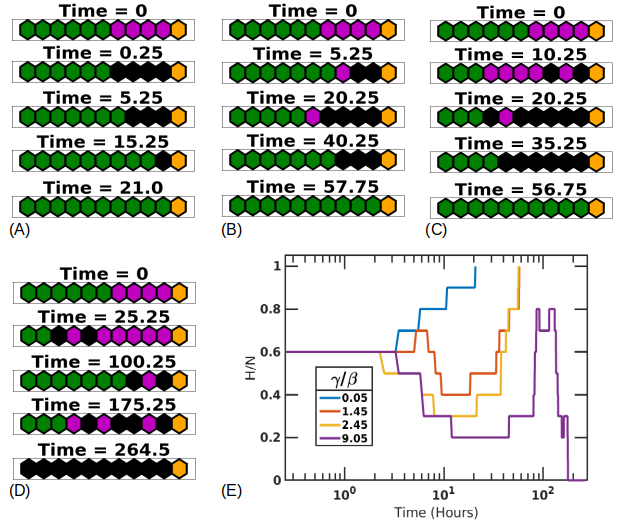

Supplement: S1 Fig — 1D simulation outputs for fixed values of β = 5 (hours). All of the figures are for fixed α/β = 1and γ/β = 0.05 (A), 1.45 (B), 2.45 (C) and 9.05 (D) with trajectories shown in (E). (A),(B) lie to the left of line demarcating γ/β* = 2.25±0.37 and have more than 0.95 probability of regaining all the healthy cells. (TIF) [file pone.0243451.s001.tif]

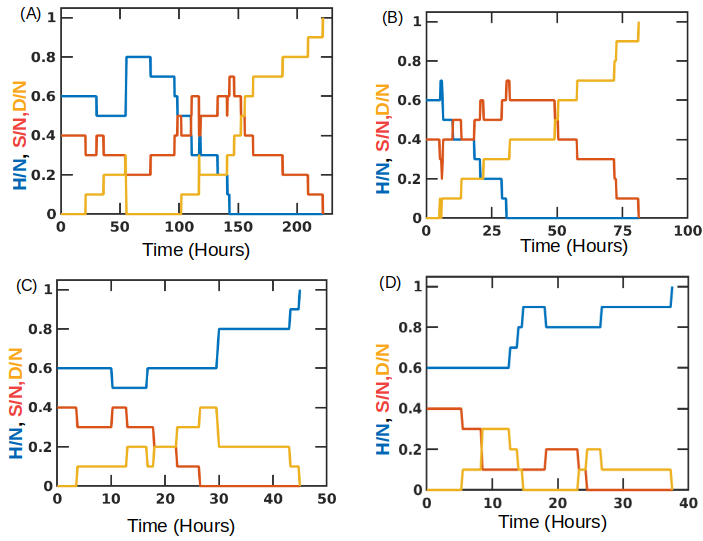

Supplement: S2 Fig — Trajectories for a small (fast) proliferation timescale α/β = 0.05 for two different trials (A,B) and a larger (slower) proliferation timescale of α/β = 1.05 (C,D). Both are for a large (slow) death timescale with γ/β = 8.05 and show how a fast proliferation timescale helps propagate the tissue damage pattern. (TIF) [file pone.0243451.s002.tif]

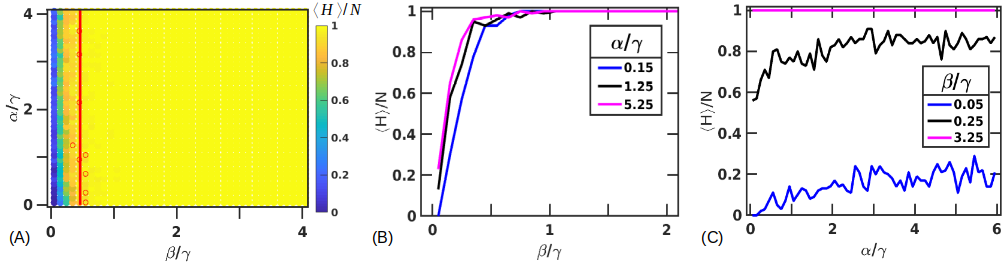

Supplement: S3 Fig — Tissue recovery probability as a function of β, α with fixed γ = 5 (hours) shows a low ratio of β/γ* = 0.47±0.07 (red line) marking the transition above which recovery probability is greater than 0.95 (A). Variation in recovery probability keeping α/γ fixed at 0.15, 1.25, 5.25 (lines in blue, black, and magenta respectively) across values of β/γ (B) and fixing β/γ = 0.05, 0.25, 3.25 (lines in blue, black, magenta respectively) across values of α/γ (C). (TIF) [file pone.0243451.s003.tif]

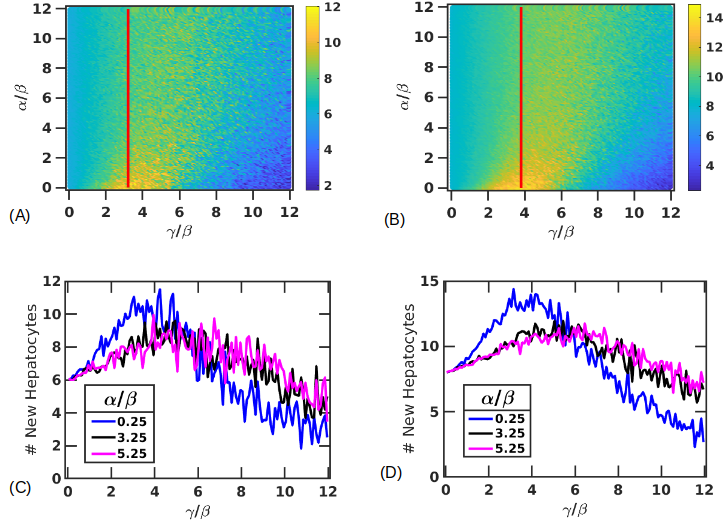

Supplement: S4 Fig — Average number of new hepatocytes (shown by the colorbars) created for fixed β = 5 (hours) for N = 16 (A,C) and N = 20 (B, D). Lines indicate γ/β* = 3.22±0.59 in (A) and γ/β* = 3.81±0.51 in (B). (C,D) shows maximum proliferation as a function of γ/β for fixed α/β = 0.25 (blue), 3.25 (black), 5.25 (magenta) for N = 16 (C) and N = 20 (D). (TIF) [file pone.0243451.s004.tif]

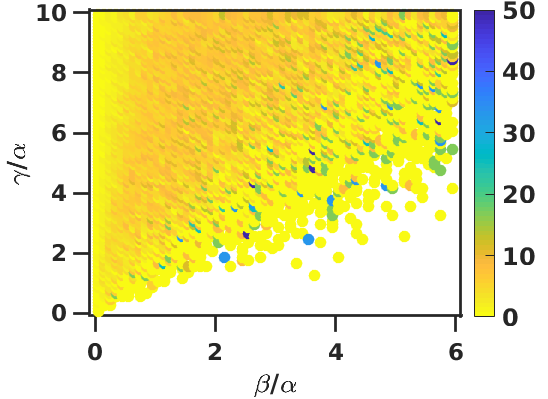

Supplement: S5 Fig — Gain of healthy cells in simulations that ultimately die. Gain percentage is calculated as (⟨max(H)⟩−Hi)/Hi*100%. In general, very few healthy cells are gained in simulations that ultimately progress to complete cell death. (TIF) [file pone.0243451.s005.tif]

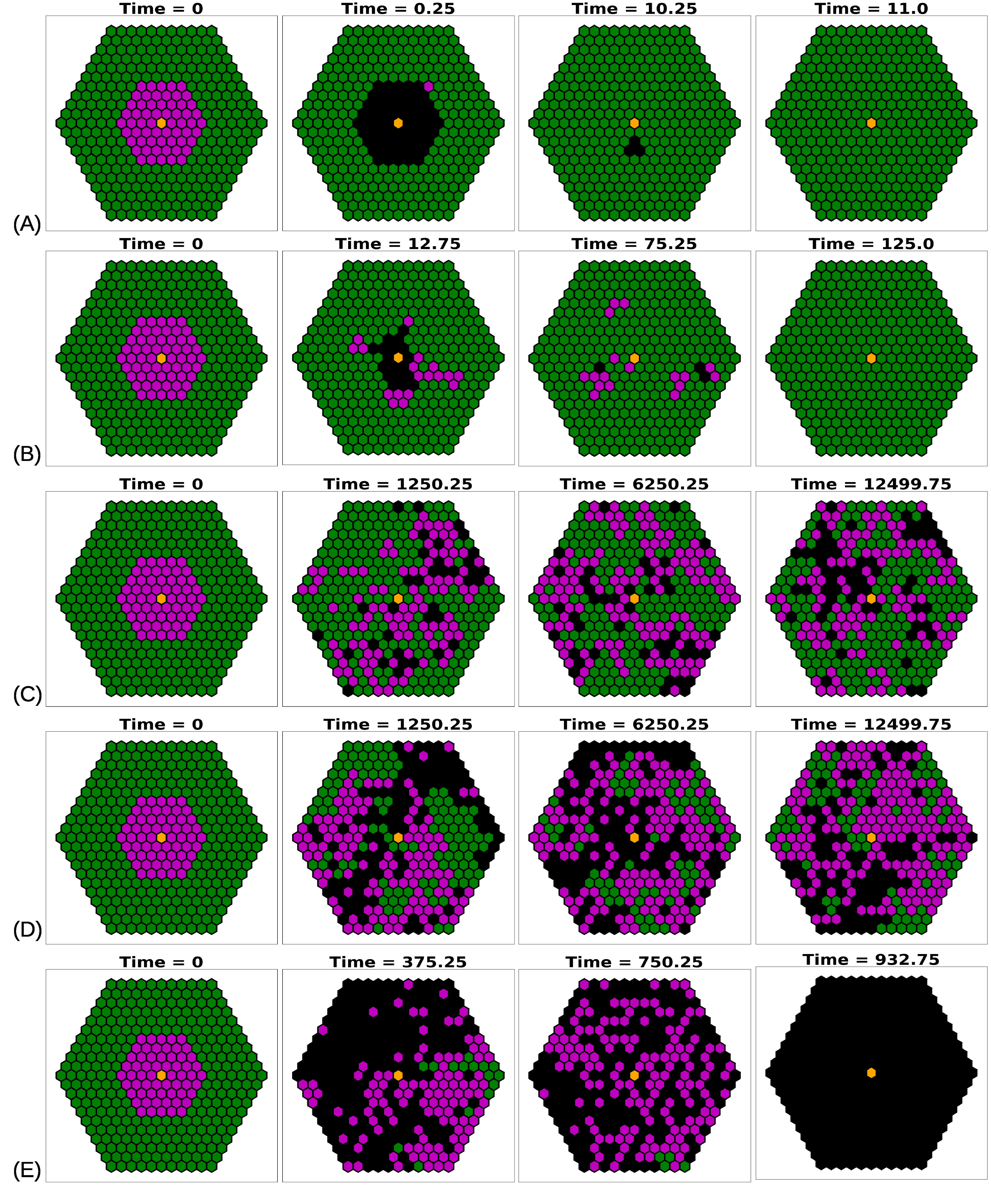

Supplement: S6 Fig — Healthy states are shown in green, stressed states in magenta and dead states in black. β is fixed at 5(hours). Output trajectories for initial conditions with 4 stressed layers with α/β = 1 and γ/β = 0.05 (A), 1.45 (B), 2.45 (C), 4.45 (D) and 9.05 (E). (TIF) [file pone.0243451.s006.tif]

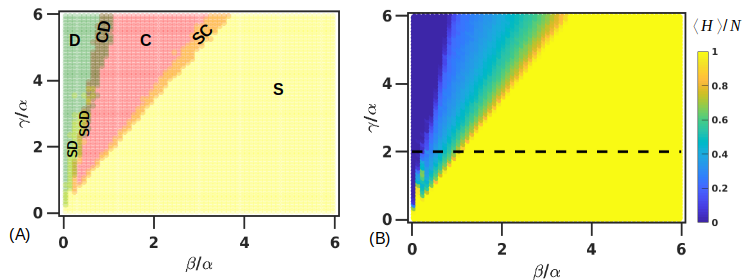

Supplement: S7 Fig — (A) Simulations on a hexagonal lobule with fixed α = 5 (hours). Coexisting states shown in red (marked with C), parameters for which the tissue always survives shown in yellow (marked with S) and regions where the tissue always dies shown in green (marked with D). Similar to the parameter space seen in Fig 8A, for ratios of β/α below 0.15 (α/β>6.7), the transition from recovery to death goes through regions of bistable outcomes of both tissue survival and death. By increasing that ratio to around β/α = 0.25 (α/β = 4), a mixture of coexistence and survival states is seen which again transitions into regions characterized by death, recovery and coexistence as γ/α is increased. As β/α is further increased (α/β is decreased) pure coexisting states can be seen wedged between different recovery and death regions. (B) Average size of the number of healthy states. Visualization outputs along the dashed line shown in S8 Fig. (TIF) [file pone.0243451.s007.tif]

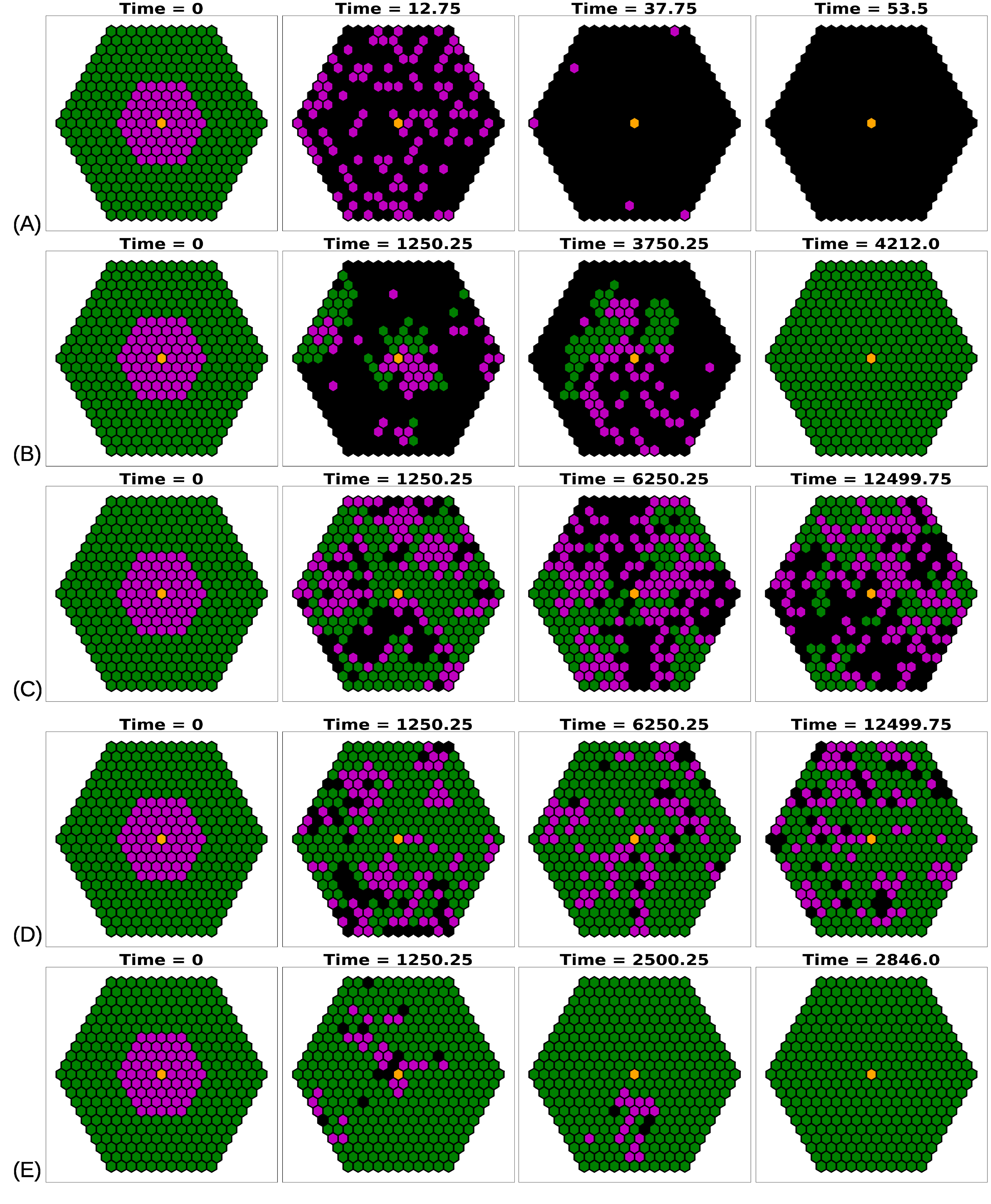

Supplement: S8 Fig — Healthy states are shown in green, stressed states in magenta and dead states in black. α is fixed at 5 (hours). Output trajectories for initial conditions with 4 stressed layers for γ/α = 2 with β/α = 0.05 (A). Here a small β results in converting healthy cells to stressed cells quite easily and the tissue dies. On increasing the ratio of β/α = 0.3, the tissue can show signs of survival (B). As the ratio is increased, tissue shows signs of coexistence β/α = 0.55 in (C) and β/α = 0.95 in (D) with different population levels of (H, S, D). For larger ratios, β/α = 1.15 in (E), the tissue shows signs of survival again. (TIF) [file pone.0243451.s008.tif]

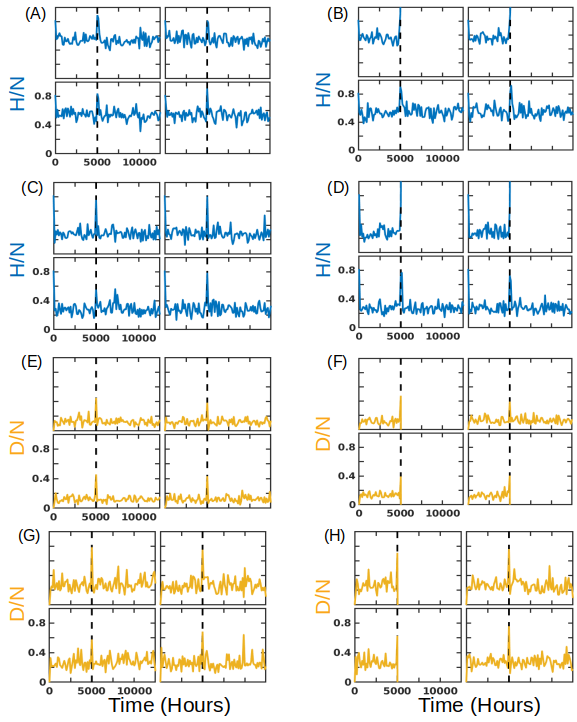

Supplement: S9 Fig — Sample trajectories with the perturbation S−>H in (A-D) and S−>D (E-H) after the system has reached the steady coexistence state. Dotted lines indicates time of applied perturbation. Each panel consists of different trials. (A,B,E,F) are with α/β = 1, γ/β = 2.45 and (C,D,G,H) are with α/β = 1, γ/β = 4.45. All the values in the left column are when 95% of stressed cells are converted into the healthy or dead states while the right column shows output when all but one stressed cell remains. Coexisting states are stable and the perturbations die down even after 95% removal of the stressed cells. (S1 Table). (TIF) [file pone.0243451.s009.tif]

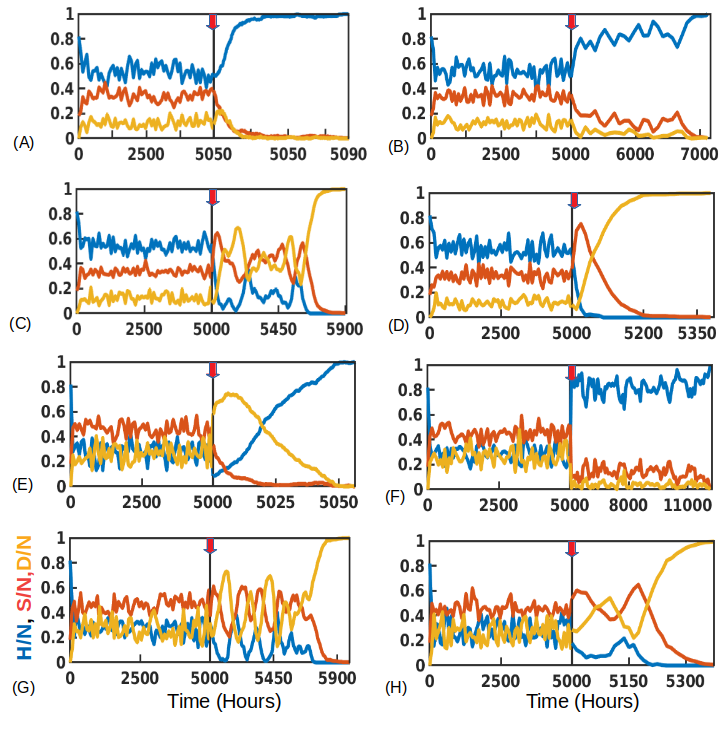

Supplement: S10 Fig — Initial parameters lead the system to a stable coexisting population after which the parameters are changed. (A-D) α/β = 1, γ/β = 2.45. At Time=5000.25 (hours) marked by the red arrows, γ/β is changed to 0.05 (A), 1.85 (B), 9.05 (C), 10.45 (D). (E-H) has α/β = 1, γ/β = 4.45 where γ/β is changed to 0.05 (E), 1.85 (F), 9.05 (G), 10.45 (H). System goes to the expected outcome at the new parameters. (TIF) [file pone.0243451.s010.tif]

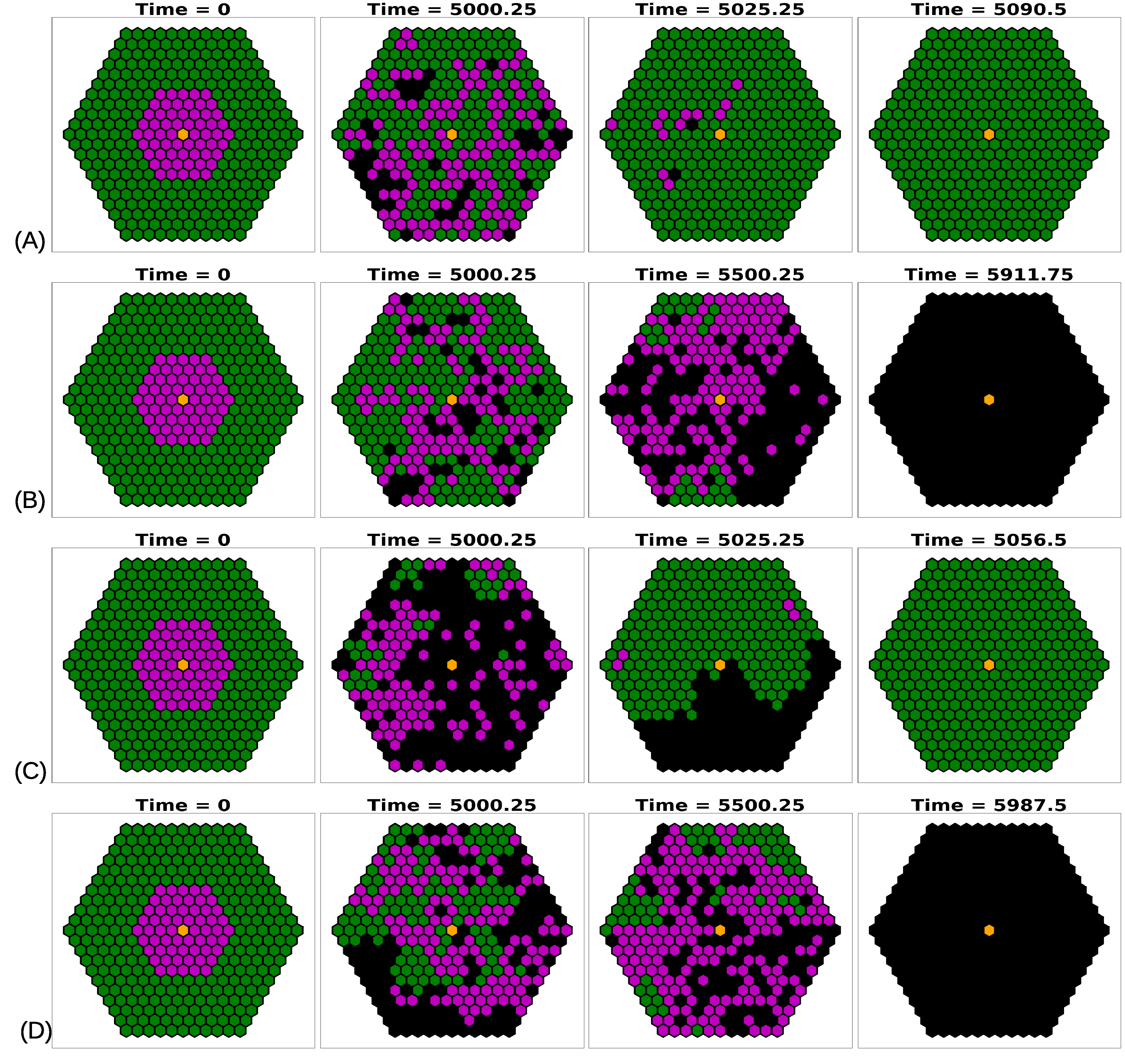

Supplement: S11 Fig — (A-B) has γ/β = 2.45 and a shift to 0.05 (A) and 9.05 (B) at Time=5000.25 (hours). (C-D) is with γ/β = 4.45 and a shift to 0.05 (C) and 9.05 (D) at Time=5000.25 (hours). (TIF) [file pone.0243451.s011.tif]

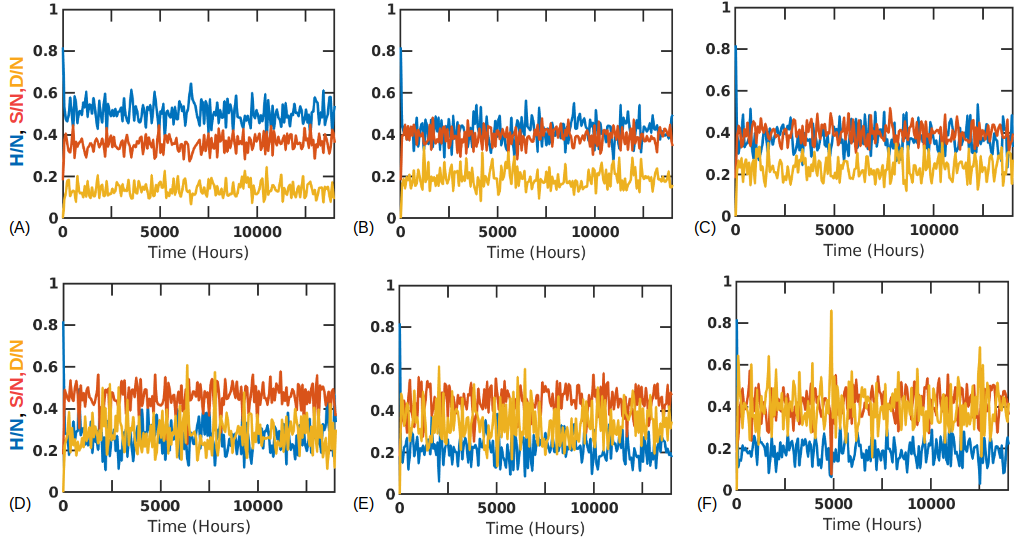

Supplement: S12 Fig — Conversion rule with an additional small probability ϵ of making a healthy cell stressed even in the absence of other stressed cells for γ/β = 2.45 (A-C) and γ/β = 4.45 (D-F). Healthy cell fraction decreases with ϵ = 0.01 (A,D), 0.05 (B,E), 0.1 (C,F) as compared to ϵ = 0 in Fig 8E and 8F. (TIF) [file pone.0243451.s012.tif]

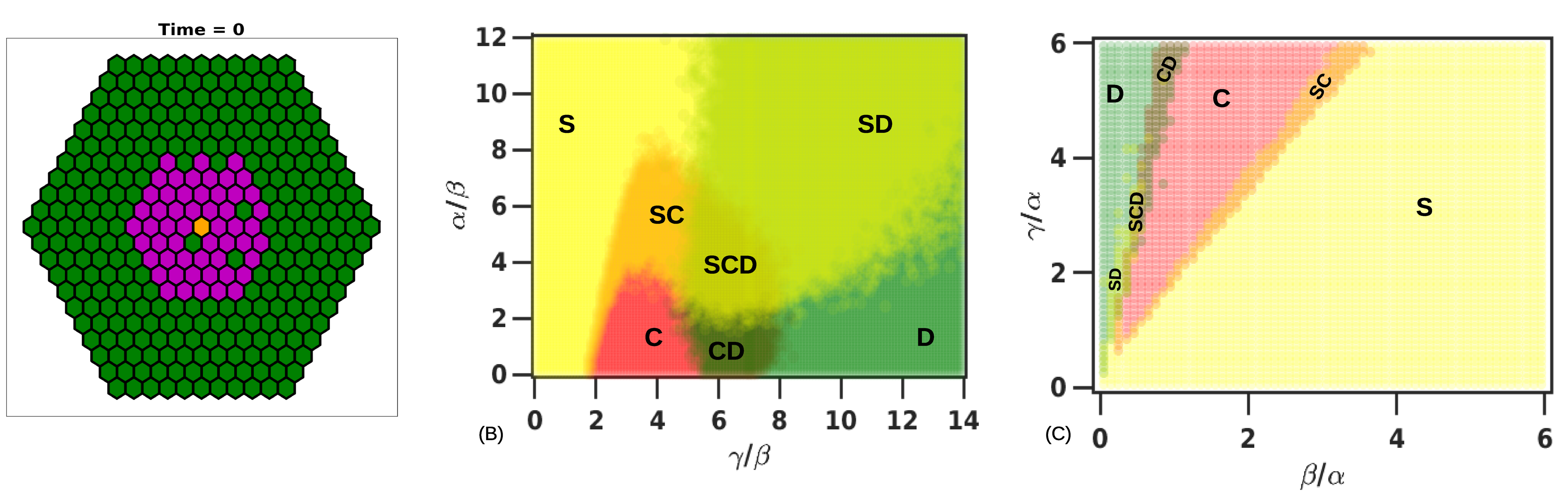

Supplement: S13 Fig — (A) 2D initial conditions where cells in the four nearest layers next to the CV have 80% probability of being in a stressed state. (B) 2D state space with the modified initial conditions; β = 5 (hours); survival states are marked with S and colored in yellow, dead states are marked with D and colored in green and coexistence states are colored in red. (C) 2D state space with fixed α = 5 (hours). Phase space outcomes are similar to Fig 8A. (TIF) [file pone.0243451.s013.tif]

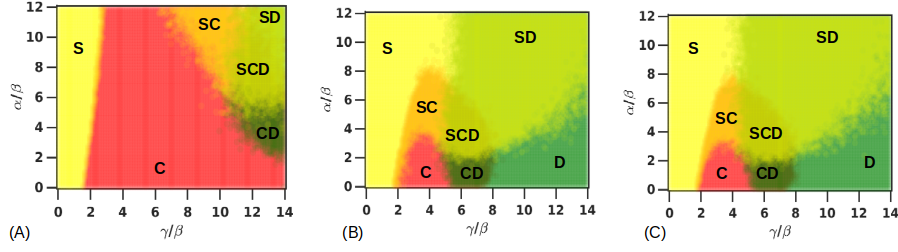

Supplement: S14 Fig — Simulations with a larger grid size with CV-PT length (NL) of 50 (N = 7650) cells over 20 trials (A). Expansion of the coexistence region is seen. Different initial conditions with a fixed NL = 10, N = 330 and number of stressed cell layers at 2 (B) and 8 (C) along the CV-PT axis. The SD boundary shifts to the left in (C) similar to the 1D results, but the coexistence region shows little changes. (TIF) [file pone.0243451.s014.tif]

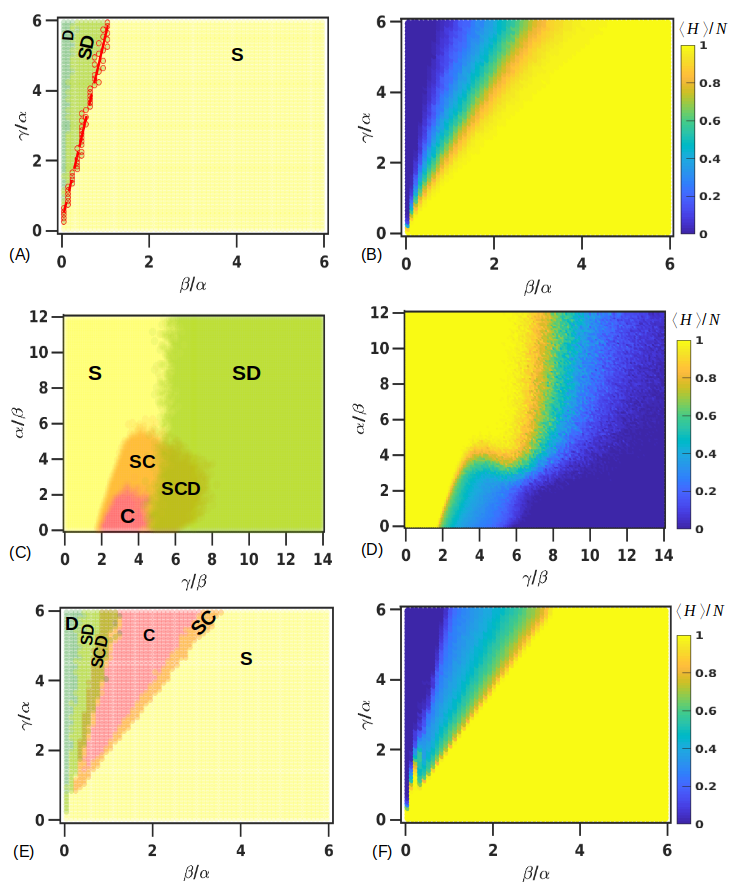

Supplement: S15 Fig — Space with maximum number of allowed divisions at 1, (A) shows possible outcomes as a phase space (B) shows the average healthy population at the end of the simulation. (C-F) Increasing the allowed number of divisions per hepatocyte to 2 brings back the coexisting states. Parameters are for fixed β = 5(hours) (C-D); fixed α = 5 (hours) (E-F). (TIF) [file pone.0243451.s015.tif]

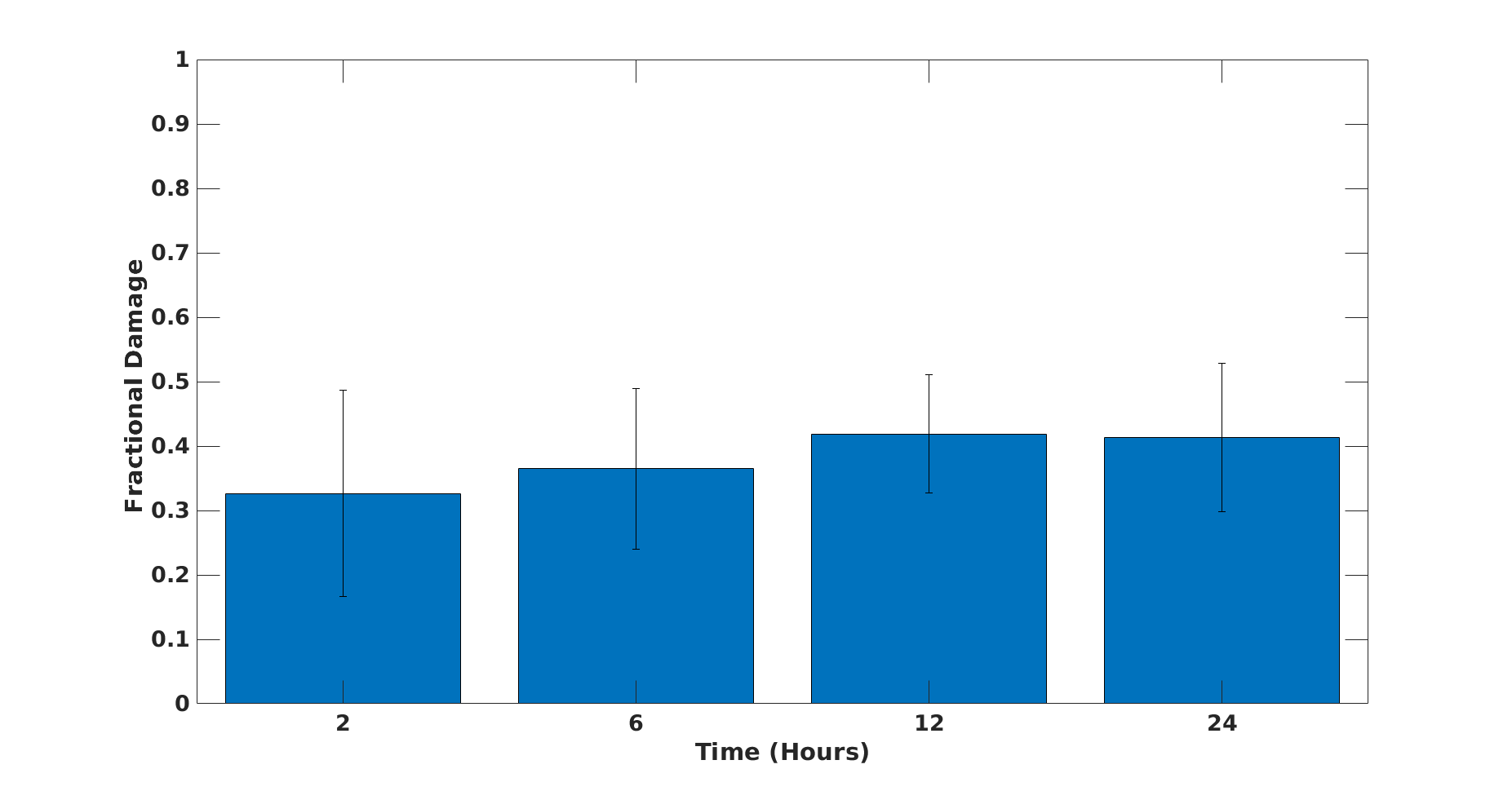

Supplement: S16 Fig — Fig shows the fractional damage along the CV to PT axis at 2, 6, 12 and 24 hours for an APAP dose of 250 mg/kg. Damage regions are relatively unchanged. (TIF) [file pone.0243451.s016.tif]

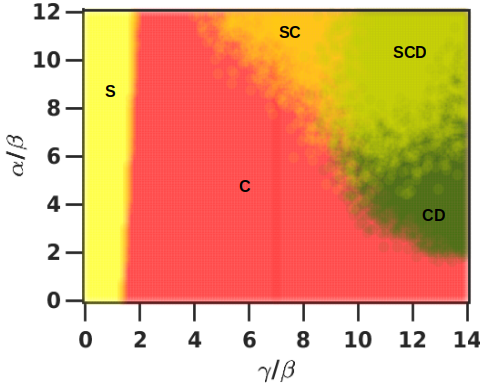

Supplement: S17 Fig — 3D simulations with 10 layers along a vertical z-axis, each consisting of hexagonal 2D lattice. Each cell in a hexagonal plane can access the neighboring site to the top and bottom planes, making the total number of nearest neighbors to 8. Simulations were done over 25 trials. Significant expansion of the coexisting regions are seen as compared to Fig 8A. (TIF) [file pone.0243451.s017.tif]
