# Supplementary material for: A computational model of liver tissue damage and repair
Source: PLoS One. 2020 Dec 21;15(12):e0243451. doi: 10.1371/journal.pone.0243451 (PMC7752149; doi:10.1371/journal.pone.0243451)
Supplement: S1 Table — (DOCX) [file pone.0243451.s018.docx]

**S1 Table. Probabilities of tissue survival on applying different perturbations at steady state**

|  | **Type of Perturbation** | | | |
| --- | --- | --- | --- | --- |
|  | $S->H$ | | $S-> D$ | |
| $\gamma\boldsymbol{/\beta}$ | **0.95*S** | **S-1** | **0.95*S** | **S-1** |
| **2.45** | 0.01 | 0.49 | 0.05 | 0.61 |
| **4.45** | 0 | 0.30 | 0 | 0.42 |

Table shows different perturbations applied to the steady state stressed cell populations. Perturbations usually die out, but probabilities of tissue recovery are greater if stressed cells die ($S-> D$) rather than being converted to healthy cells ($S->H$).
