# Supplementary material for: A computational model of liver tissue damage and repair
Source: PLoS One. 2020 Dec 21;15(12):e0243451. doi: 10.1371/journal.pone.0243451 (PMC7752149; doi:10.1371/journal.pone.0243451)
Supplement: S1 File — (ZIP) [file pone.0243451.s019.zip › supplementary_doc_codes/Supporting Information.docx]

# A computational model of liver tissue damage and repair

Priyom Adhyapok^1,2*^, Xiao Fu^3^, James P. Sluka^1,4^, Sherry G. Clendenon^1,4^, Victoria D. Sluka^1,#a^, Zemin Wang^5,#b^, Kenneth Dunn^6^, James E. Klaunig^5^, James A. Glazier^1,4^

^1^ Biocomplexity Institute, Indiana University, Bloomington, IN, United States of America.

^2^ Department of Physics, Indiana University, Bloomington, IN, United States of America.

^3^ The Francis Crick Institute, London, United Kingdom.

^4^ Department of Intelligent Systems Engineering, Indiana University, Bloomington, IN, United States of America.

^5^ School of Public Health, Indiana University, Bloomington, IN, United States of America.

^6^ School of Medicine, Indiana University, Indianapolis, IN, United States of America.

^#a^ Current Address: Institute of Archaeology, University College London, London, United Kingdom

^#b^ Current Address: Food and Drug Administration, Washington D.C., United States of America

*Corresponding author

E-mail: priyom.adhyapok@gmail.com (PA)

1D Parameter space outcomes for fixed $\gamma$

We fix $\gamma=5$(hours) and vary the parameters $\alpha,\beta$. This leads to similar outcomes where we now see the presence of the bistable states for low values of $\beta/\gamma$ (high $\gamma/\beta$) (S3A Fig). A critical ratio of $\beta/\gamma^{*}=0.47\pm0.07$ (red line in S3A Fig) is obtained by tracking points where the survival probability falls to 0.95. We can now also see values for which the tissue completely dies for low $\beta/\gamma=0.05$. For low values of $\beta/\gamma$ (large $\gamma/\beta$), only a large proliferation timescale can achieve survival, and then only in a few simulations (blue curve in S3C Fig). Additional curves in S3C Fig are plotted for $\beta/\gamma=0.25, 3.25$ (black and magenta respectively) and show the survival probability as a function of $\alpha/\gamma$. The curves in S3B Fig are plotted for horizontal lines across $\beta/\gamma$ for fixed $\alpha/\gamma=0.15, 1.25, 5.25$.

## Perturbations to the coexisting states

We check the stability of the coexisting states by applying perturbations to the steady state populations to check if the tissue can be pushed towards recovery. We let the system evolve to the coexisting populations, then change one of the population levels, namely the stressed cells in this case, to either a healthy or the dead state and observe what the new values are. We check for two specific cases where $95\%$ of the stressed population is converted to the healthy or dead states and when every stressed cell except one is converted to these states. We simulated these perturbations over 100 different trials for different values of $\gamma/\beta=2.45, 4.45$ in the coexistence region and also measured the probabilities of the tissue surviving (S1 Table). S9 Fig shows the output trajectories for conversion of stressed cells to healthy ones. Dashed line indicates the time of the application of the perturbation.

S9A Fig shows the healthy population fractions with the perturbation applied to $\gamma/\beta=2.45$ for four different trials where 95% of the stressed cells are converted to healthy ones. We observe that the perturbations quickly die down and the system reverts back to the steady state population for that parameter combination. The probability of the perturbation making an effect on the system and leading the tissue to recovery is extremely low (0.01, S1 Table). On converting every stressed state except for 1 in S9B Fig, we find some trajectories leading to a complete healthy population. However, the majority of the simulations (51%) still reach the old coexistence state if the remaining stressed cell is able to affect any other cells without disappearing. We see a similar behavior for $\gamma/\beta=4.45$ where 95% of the conversions in S9C Fig doesn’t change the behavior of the system and removing all but one stressed cell (S9D Fig) has 30% probability of reaching survival. We note that $\gamma/\beta=4.45$ has a lower probability of the perturbation surviving than $\gamma/\beta=2.45$. This can be attributed to the larger value of $\gamma$ contributing to a longer timescale of clearance and death.

S9E-S9H Figs shows a few sample trajectories after converting the stressed cells into dead ones. S9E-S9F Figs show D/N for $\gamma/\beta=2.45$ and S9G-S9H Figs have $\gamma/\beta=4.45$ with 95% of the stressed cells removed as dead ones (S9E and S9G Figs) and all but one stressed cell removed in (S9F and S9H Figs). On measuring the probabilities, we find that overall the perturbation for $S->D$ are more successful than $S->H$ (S1 Table).

However, in general these results indicate that any perturbations to the coexistence states are very likely to die out.

**S9 Fig. Perturbations to the steady state populations**

Sample trajectories with the perturbation $S-> H$ in (A-D) and $S-> D$(E-H) after the system has reached the steady coexistence state. Dotted lines indicates time of applied perturbation. Each panel consists of different trials. (A,B,E,F) are with $\alpha/\beta=1, \gamma/\beta=2.45$ and (C,D,G,H) are with $\alpha/\beta=1, \gamma/\beta=4.45$. All the values in the left column are when 95% of stressed cells are converted into the healthy or dead states while the right column shows output when all but one stressed cell remains. Coexisting states are stable and the perturbations die down even after 95% removal of the stressed cells. (S1 Table )

**S1 Table. Probabilities of tissue survival on applying different perturbations at steady state**

|  | **Type of Perturbation** | | | |
| --- | --- | --- | --- | --- |
|  | $S->H$ | | $S-> D$ | |
| $\gamma\boldsymbol{/\beta}$ | **0.95*S** | **S-1** | **0.95*S** | **S-1** |
| **2.45** | 0.01 | 0.49 | 0.05 | 0.61 |
| **4.45** | 0 | 0.30 | 0 | 0.42 |

Table shows different perturbations applied to the steady state stressed cell populations. Perturbations usually die out, but probabilities of tissue recovery are greater if stressed cells die ($S-> D$) rather than being converted to healthy cells ($S->H$).

We also check if the final output is dependent on the nature of the patterns in the coexistence state. For these simulations, we wait for the system to reach the steady coexistence state, then change the parameter values to a value that is either in the survival or death region. We fix our $\alpha/\beta=1$. S10A-S10D Figs show trajectories with initial condition with $\gamma/\beta=2.45$ which reach a steady coexistence state with a higher healthy population. At Time= 5000.25 (hours) (indicated by the red arrows), the ratio $\gamma/\beta$ is changed to 0.05 (A), 1.85 (B), 9.05 (C), 10.45 (D). In all the cases the final behavior is characteristic of the output for that region of parameter space. Trajectories closer to the coexistence region ($\gamma/\beta=1.85, 9.05$) show oscillations in the population trajectories as they progress to the different steady state, as compared to parameters farther away ($\gamma/\beta=0.05, 10.45$).

S10E-S10H Figs show trajectories with initial condition with $\gamma/\beta=4.45$ which reach a steady coexistence state with a lower healthy population. At Time= 5000.25 (Hours), the ratio $\gamma/\beta$ is similarly changed to 0.05 (E), 1.85 (F), 9.05 (G), 10.45 (H). Again regardless of the initial state of the system, the final behavior is characteristic of the output for that region of parameter space.

**S10 Fig. Perturbation to the model parameters**

Initial parameters lead the system to a stable coexisting population after which the parameters are changed. (A-D) $\alpha/\beta=1,\gamma/\beta=2.45$. At Time=5000.25 (hours) marked by the red arrows, $\gamma/\beta$ is changed to 0.05 (A), 1.85 (B), 9.05 (C), 10.45 (D). (E-H) has $\alpha/\beta=1, \gamma/\beta=4.45$where $\gamma/\beta$is changed to 0.05 (E), 1.85 (F), 9.05 (G), 10.45 (H). System goes to the expected outcome at the new parameters.

Some of the visualization output is shown in S11 Fig with (A-B) with $\gamma/\beta=2.45$ and a shift to

0.05 (A) and 9.05 (B). (C-D) is with $\gamma/\beta=4.45$ and a shift to 0.05 (C) and 9.05 (D).

**S11 Fig. Visualization output from the model parameter perturbations**

(A-B) has $\gamma/\beta=2.45$ and a shift to 0.05 (A) and 9.05 (B) at Time=5000.25 (hours). (C-D) is with $\gamma/\beta=4.45$ and a shift to 0.05 (C) and 9.05 (D) at Time=5000.25 (hours)

## Additional Parameter Space With Fixed Number of Divisions

We additionally plot the possible number of states for fixed $\alpha=5$ (hours) for the maximum number of allowed hepatocyte divisions fixed at 1 (S15A Fig). As seen in Fig 9D, transitions from survival to bistable regions can be seen. Line shown in red marks the transition for $\gamma/\beta=5.75\pm1.10$. Additionally, this space points to values of large $\gamma$ for small $\beta/\alpha$ (large $\alpha/\beta$) for which the tissue shows complete signs of hepatocyte death (characterized by D) and which was unexplored in Fig 9D. We note again that in this case, any final state with any remaining healthy states will be classified under ‘S’, so the average number of healthy states for the parameters shown in S15B Fig gives us a better idea of what the extent of the damage is.

We also find that features of the state space similar to Fig 8A can now be retrieved by increasing the maximum number of allowed divisions to 2 for each hepatocyte (S15C Fig, S15E Fig). We note that the line demarcating the transition from survival (S) to bistability (SD) occurs around the same ratio of $\gamma/\beta\approx5$ with the coexistence states now superimposed on the space. Average healthy cell fractions shown in S15D Fig, S15F Fig.

**S15 Fig. Parameter spaces with fixed number of divisions**

Space with maximum number of allowed divisions at 1, (A) shows possible outcomes as a phase space for 100 different trials (B) shows the average healthy population at the end of the simulation.

(C-F) Increasing the allowed number of divisions per hepatocyte to 2 brings back the coexisting states. Parameters are for fixed $\beta=5$ (hours) (C-D); fixed $\alpha=5$ (hours) (E-F).
